# Time to coronary angiography and revascularization in 575,247 patients with STEMI from 2012 to 2023: a retrospective population-based cohort study

**DOI:** 10.1016/j.lanepe.2025.101576

**Published:** 2025-12-29

**Authors:** Paulina E. Stürzebecher, Ulrich Laufs, Philip Baum, Johannes Diers, Armin Wiegering, Konstantin Uttinger

**Affiliations:** a Klinik und Poliklinik für Kardiologie, Universitätsklinikum Leipzig, Leipzig, 04103, Germany; b Department of Thoracic Surgery, Thoraxklinik at Heidelberg University Hospital, Röntgenstraße 1, 69126, Heidelberg, Germany; c Marienkrankenhaus Hamburg, Alfredstraße 9, 22087, Hamburg, Germany; d Department of General, Visceral, Transplant and Thoracic Surgery at Frankfurt University Hospital, Goethe University, Frankfurt am Main, Germany; e Frankfurt Cancer Institute, Georg-Speyer-Haus, Paul-Ehrlich-Str. 42-44, 60596, Frankfurt am Main, Germany; f University Cancer Center Frankfurt (UCT), Frankfurt University Hospital, Goethe University, Frankfurt am Main, Germany

**Keywords:** STEMI, In-hospital mortality, In-hospital time to angiography, In-hospital delay, Geographic routing, Administrative data, Routine data

## Abstract

**Background:**

Rapid primary percutaneous coronary intervention (PPCI) in patients with ST-elevation myocardial infarction (STEMI) reduces in-hospital and long-term mortality. This study analyzes time intervals to PPCI in STEMI, risk factors for delay of PPCI, and in-hospital mortality from 2012 to 2023.

**Methods:**

This is a retrospective population-based analysis of hospital billing data of adult STEMI patients receiving PPCI in Germany. The time for transport to hospital (TTH) was estimated using geographic routing. The in-hospital time to angiography (IHTA) was calculated using time coding of PPCI in patient records.

**Findings:**

A total of 575,247 patients were included. The median age was 64 years, 28.5% (164,016) were female. The population with IHTA ≤60 min increased from 44.5% (22,240/49,965) in 2012 to 57.7% (24,434/42,356) in 2023 with improved TTH + IHTA ≤120 min (56.6%, 28,280/49,965, in 2012–70.2%, 29,734/42,356, in 2023). IHTA improved from median 73.1 min (IQR 25.2–186.6) in 2012 to 46.4 min (IQR 17.5–111.6) in 2023 with a stable TTH (11.4–11.9 min). Risk factors for an IHTA >60 min included age, female sex, comorbidity, presentation out of regular hours, and low-volume hospitals. In-hospital mortality increased (8.8%, 4406/49,965, in 2012, 10.4%, 4822/46,203, in 2021, 10.1%, 4272/42,356, in 2023), paralleling a rise in patient age and comorbidity. Risk factors for in-hospital mortality included female sex, increased age, comorbidity, high-volume hospitals, intervention of multiple coronary arteries, weekend admission, and presentation out of regular hours. IHTA <40 min (90–120 min as reference) and TTH + IHTA <80 min (≥120 min as reference) reduced the risk of death.

**Interpretation:**

Combining hospital billing records with geographic routing enables benchmarking of both pre- and in-hospital delays in STEMI care. In hospital delay decreased between 2012 and 2023. Important areas for improving time delays and STEMI-related mortality include the timeliness of care outside of regular hours and a focus on women, older patients, as well as individuals with comorbidities.

**Funding:**

There was no funding for this project or this publication.

**Research in context**

**Evidence before this study**

PubMed and MEDLINE were searched for existing evidence using the search terms “myocardial infarction”, “STEMI”, or “ST-elevation myocardial infarction”, and “time to/timing of/delay of/delayed diagnostic/revascularization/coronary angiography/coronary diagnostic”, which was last conducted on January 1st 2025. Only original clinical studies were considered, except small (<500 cases) monocentric retrospective analyses. The resulting studies and their references were screened for relevance. Current evidence shows that acute ST-segment elevation myocardial infarction remains one of the most time-critical cardiovascular emergencies. Numerous studies have demonstrated that timely mechanical reperfusion via PPCI is crucial to improve outcome, e.g. to reduce infarct size, heart failure, and mortality. In an overall effort to minimize ischemic time, door-to-balloon time is proven to be an independent factor associated with reduced short-term and long-term (beyond 6 months) mortality if it is shorter than 90 min, while further shortening of this interval might correlate with further reduction of mortality. During the past 30 years, reducing in-hospital delay became a priority; several strategies to reduce in-hospital time to PPCI were identified and implemented, resulting in significant decline of delay.

**Added value of this study**

This real-world large-scale analysis of population-based in-hospital billing data combined with a geographic routing approach in STEMI patients introduces an updated benchmarking of in-hospital time to angiography in Germany, enabling future automated quality reporting. A risk factor analysis for in-hospital delay and in-hospital mortality underscored critical areas for improvement: enhancing care during out-of-hours presentations and ensuring guideline-recommended time targets across sexes. Additionally, elderly and comorbid patients were found to be at increased risk for both treatment delays and adverse outcomes.

**Implications of all the available evidence**

While a reduction in treatment delay in STEMI care has been achieved, inequalities in patient care persist, especially with regards to impaired care in females. The elderly and comorbid remain at risk of suboptimal care, enabling STEMI-networks to focus on these patient groups.

## Introduction

The time to reperfusion via primary percutaneous coronary intervention (PPCI) improves outcomes of patients with ST-segment elevation (STEMI).^1^ Every minute of ischemic time in STEMI affects 1-year mortality; the door-to-balloon time ≤90 min is an independent predictor of reduced short-term and long-term mortality and further reductions in this interval may correlate with additional mortality benefits.^2^, ^3^, ^4^, ^5^, ^6^ Over the past three decades, reducing in-hospital delays became a priority of public health policies. Strategies such as pre-hospital electrocardiograms and emergency room bypass have led to substantial improvements.^7^, ^8^, ^9^, ^10^, ^11^

Based on this evidence, the 2023 ESC Guidelines highlight three specific time targets: a total ischemic time of ≤120 min, with a ≤90-min goal from first medical contact to wire crossing, and an in-hospital delay of ≤60 min.^12^ To implement the guidelines, regular benchmarking is imperative to identify factors contributing to delays in STEMI treatment. Identifying these delays, whether pre-hospital or during in-hospital processes, and understanding associated factors, are essential for effective quality improvement initiatives.

This study aims to examine current trends in in-hospital time to angiography (IHTA) and time of transport to hospital (TTH) in STEMI care in Germany, aligned with guideline-recommended timeframes. Furthermore, we seek to identify patient- and system-level factors linked to delays and mortality, exploring modifiable determinants at a cohort level to guide optimization strategies for regional STEMI networks. We applied the novel method of combining administrative data for IHTA as surrogate parameter for door-to-balloon time combined with geographic routing computation approach for TTH to provide insights into STEMI patient care in Germany.

## Methods

### Data source

This retrospective, population-based cohort study utilizes anonymized billing data provided by the "Statistische Bundesamt” (Federal Statistical Office in Germany). The data source includes the RDC of the Federal Statistical Office and Statistical Offices of the Federal States (DOI: 10.21242/23141.2012.00.00.6.1.0–10.21242/23141.2023.00.00.6.1.0 and 10.21242/23141.2019.00.00.6.1.1, own calculations). Data acquisition was conducted in collaboration with the Federal Statistical Office, adhering to their guidelines for handling highly sensitive patient-record data. No ethical approval was required for this large-scale, nationwide cohort analysis. All billing records from hospital admissions in Germany are registered annually with the “Statistische Bundesamt,”. Patients for this study were identified using coding according to the International Classification of Diseases (ICD-10, German Modification, GM), ICD coding, and OPS, “Operationen und Prozedurenschlüssel,” procedural coding.

### Study population

All adult patient records admitted for ST-Elevation Myocardial Infarction (STEMI) as the primary diagnosis (I21.0, I21.1, I21.2, I21.3) between January 1, 2012, and December 31, 2023, with at least one coded coronary angiography (OPS 1–275) and no lysis therapy (OPS 8–020.8) were included (Supplementary Table S1, Supplementary Figure S1). In case of duplicates based on all available variables, one record was randomly kept for further analysis. Transferred patient records with a coded previous length of stay longer than 24 h at the initial treating hospital were excluded. For identification of availability of Coronary-Bypass-surgery at center level, patient records with a performed Coronary Bypass were initially included, but were excluded for further analysis. Each patient record contained data on age, sex, an anonymized institute identifier, procedural codes (OPS) including date and time, primary and secondary diagnoses, length of stay, and reasons for admission and discharge. Only the index hospitalization was available for all analyses; no patient identifier was available, precluding the identification of readmissions of individual patients. In accordance with defined outcome measures, different sub-cohorts were pre-specified to be analyzed separately: the total cohort and sub-cohorts excluding patient records with an interval of in-hospital time to angiography (IHTA) > 120 min (for all analyses regarding in-hospital mortality).

### Time intervals of interest: In-hospital time to angiography, and transport time to hospital obtained using geographic routing

The time of admission to the hospital was recorded with the exact time and date. While the coded date and its plausibility are confirmed during the billing process, the time of admission is subject to variability (actual time of admission vs time of reading the health chip card). There is a linked time variable with a specific affiliation to an individual OPS code. These time variables reflect the beginning of the OPS procedure as specified in the billing data process (according to § 21 KHEntgG, German “Krankenhausentgeltgesetz”). It is not verifiable if this, in the case of angiographies, is the time of preparations, the time of puncture of the artery, the time of stenting/ballooning, or the documentation of the procedure code, as coding varies among hospitals. Implausible coding of the time from admission to coronary angiography in STEMI has been reported to be in the range of 8%^13^; records with implausible coding (negative interval) were excluded from this analysis. The interval of time from coded admission to coded coronary angiography is referred to as IHTA in this analysis. The IHTA cannot be equated with door-to-balloon or door-to-wire time intervals but can only be interpreted as a surrogate parameter for in-hospital delay from admission to coronary angiography. The travel distance and travel duration from the center of the patient's area code to the center of the area code of the treating hospital and to all surrounding STEMI treating hospitals hospital in a real-world setting of traffic using the most direct were obtained using geographic routing based on latitude and longitude information retrieved from Open Source Routing Machine © and OpenStreetMap ©^14^, ^15^, ^16^, ^17^; travel distance was defined as transport to hospital, TTH. Patient records with a failure of geographic routing (with no information on the patient's area code and/or the area code of the treating hospital) were excluded. For patients presenting to a hospital while not at their domicile, TTH was expected to be unplausible. Patient records with a TTH >150 min, i.e. the 99th percentile of overall TTH, were excluded due to non-plausibility. The data records contained no information on the actual route of presentation, i.e. self-presentation or by emergency medical personnel, unless the patient was admitted from another hospital, in which case no information on the previously treating hospital was available; these transferred patients were excluded from analyses regarding the combined time interval (TTH + IHTA). TTH + IHTA was used as surrogate for total delay.

### Primary and secondary outcome measures

The primary outcome measures of this analysis were the fraction of IHTA ≤60 min, the fraction of TTH + IHTA ≤120 min, the risk of IHTA >60 min, the risk of TTH + IHTA >120 min, and in-hospital mortality. Secondary outcome measures included IHTA, presented as median and interquartile range (IQR) per year over the study period, stratified by patient factors and medical system factors. Patient records with IHTA >120 min were pre-defined to be excluded for the analysis regarding in-hospital mortality to avoid heterogeneity in treatment strategies; this time window has been chosen since it corresponds to the recommended overall delay, after which a potential advantage of primary percutaneous coronary intervention over fibrinolysis may be lost.^12^^,^^18^ Post-hoc analyses included a linear regression model to analyze the impact of covariates on time intervals of interest. Additionally, post-hoc analyses were determined to investigate hospital skipping (defined as the treating hospital not being the closest STEMI treating hospital) and hospital volume–performance associations. A model investigating the re-allocation of patients to closer hospitals was pre-defined to analyze its impact on patient allocation to “treatment within 90, 90–120, and >120 min” of TTH + IHTA. All cases were assigned to hospital tertiles treating an equal number of cases during the study period (i.e. one third of patients), resulting in an assignment to a hospital tertile for each hospital (low, medium, high volume).

### Statistical analysis

Odd's ratios (OR) were calculated between independent variables and the dependent variables IHTA exceeding 60 min, TTH + IHTA exceeding 120 min, and in-hospital mortality. In a binomial multivariable mixed effect logistic regression model, the relationships between dependent and independent variables were determined. To identify covariates for the multivariable model, the “disjunctive cause criterion” based on clinical assumptions was deployed to control for available pre-exposure covariates that are potential causes of the exposure on patient level (age, sex, comorbidity, region of patient's residence) or due to staffing heterogeneities or structural discrepancies within the medical system (time of admission, weekend admission, hospital volume, location of hospital, jumper status, i.e. hospital skipping, location of hospital), or the outcome (age, comorbidity, sex, hospital performance, multiple coronary arteries intervened), or both^19^; due to unknown and complex interrelation, no causal acyclic diagram is available. We used a multivariable mixed effect linear regression model to assess the impact of covariates from the logistic regression model on time intervals (IHTA and TTH + IHTA. A multivariable fractional polynomial model was built from the initial set of continuous predictors to assess the linearity assumption using the backfitting model-selection algorithm.^20^ We set the nominal p-value for variable and fractional polynomial selection to 0.05 for non-binary variables). In all multivariable models, the treating hospital was included as random effect. Likelihood ratio tests were used to assess logistic regression model accuracy. We excluded the presence of significant multicollinearities among confounding variables based on the variance inflation factor using the Stata plugin “collin”; a variance inflation factor of 5 was pre-defined as cutoff. To determine logistic regression model discrimination performance, area under the curve results are depicted in a forest plot, and calibration plots were produced using the Stata plugin “pmcalplot” for assessment of observed vs expected probabilities of the respective dependent variable of logistic regression models. For post-hoc analyses of in-hospital mortality, the Stata plugin “margins” was used to assess the impact of the year of admission. For the logistic and linear regression model, age categories were maintained for interpretation purposes, as previously categorized in STEMI,^21^ while the linearity assumption was met for both age categories and age as continuous variable. To account for different comorbidity structures, the Charlson comorbidity index^22^ was obtained using the Stata plugin “charlson icd_sidediagnoses, index (10)”. The work has been reported in line with the STROCSS criteria^23^ and the STROBE guidelines.^24^ The Sankey diagram and maps of Germany were generated using Flourish. Graphs were generated using Prism (Version 10). Stata (Version 16; StataCorp LP, Texas, USA) was used for all statistical analyses.

### Role of the funding source

There was no funding for this project or this publication. No funder had part in study design, data collection, data analysis, data interpretation, or writing of the report.

## Results

### Patient characteristics

A total of 575,247 patient records met the inclusion criteria (Supplementary Figure S1). The incidence of STEMI declined during the observational period. The regional distribution of hospitals providing STEMI care was heterogeneous but remained stable over time (Supplementary Figure S2). The median age of patients at admission was 64 years, and increased over time (2012 mean 64.4 years, 2023 65.6 years, p<.001) (Table 1 and Supplementary Figure S3). 164,016 (28.5%) of the patients were female. The most frequent coronary status was three vessel disease (169,572; 29.5%). The most common comorbidities were arterial hypertension (336,288; 58.5%) and hyperlipidemia (257,525; 44.8%). The majority of patients (267,398; 46.5%) had a Charlson Comorbidity Index of 1, and Comorbidity increased over time (2012 mean 1.19, 2020 mean 1.31, 2023 1.23, p<.001) (Table 1 and Supplementary Figure S3). 61.7% (355,163) were admitted between 6.00 am and 4.59 pm. 137,186 (23.9%) patients were admitted on weekends. 24,407 (4.3%) patients were transferred from a different hospital. The median length of stay was 6 days (interquartile range, IQR, 4–8 days) and decreased over time (2012 mean 8.2 days, 2023 6.5 days, p<.001) (Table 1 and Supplementary Figure S3). 33.0% were treated in high volume (83 different hospitals, treating 185.8 patients in median), 33.4% in medium volume (152 different hospitals, treating 110.8 patients in median), and 33.6% in low volume tertile hospitals (644 different hospitals, treating 57.3 patients in median). 22.2% (127,742 patients) were admitted to centers with a department of cardiac surgery. The majority of patients were treated in a hospital coded as urban (46.4%, 266,794 patients). Patients’ area codes were urban in 44.9% (258,120 patients) (Table 1).

**Table 1:** Study population and patient characteristics.

Total no. of patients	575,247
**Age** (years)^a^	64.0 (55–75)
≤59 n (%)	210,802 (36.7)
60–74 n (%)	209,511 (36.4)
>75 n (%)	154,934 (26.9)
**No. of females** n (%)	164,016 (28.5)
**Sex ratio** (M:F)	2.5
**Coded coronary status** n (%)	
One vascular disease	168,917 (29.4)
Two vascular disease	151,306 (26.3)
Three vascular disease	169,572 (29.5)
Main branch disease	28,107 (4.9)
Previous bypass	5416 (0.9)
Previous coronary stenting	23,271 (4.1)
**Comorbidities** n (%)	
Arterial hypertension	336,288 (58.5)
Diabetes mellitus	131,823 (22.9)
Hyperlipidemia	257,515 (44.8)
Obesity	42,659 (7.4)
Atrial fibrillation	77,300 (13.4)
PAD	18,305 (3.2)
Chronic kidney disease	76,369 (13.3)
**Charlson comorbidity index #**n (%)	
0	214,069 (37.2)
1	267,398 (46.5)
2	70,481 (12.3)
3	23,299 (4.1)
**Time of admission** n (%)	
0.00 am–5.59 am	73,725 (12.8)
6.00 am–4.59 pm	355,163 (61.7)
5 pm–11.59 pm	146,359 (25.4)
**Weekend admission** n (%)	137,186 (23.9)
**Admission from different hospital** n (%)	24,407 (4.3)
**Length of stay** (days)^a^	6 (4–8)
**In-hospital time to angiography** (min) median (IQR), mean (SD)	57.5 (20.3–138.9), 348.7 (1692.2)
**Overall time: Transport to hospital + in-hospital time to angiography** (min) median (IQR), mean (SD)	84.4 (46.2–165.9), 373.7 (1692.1)

Values in parentheses are percentages of total in the patient group unless otherwise indicated. Coronary status does not add up to total due to 28,658 (5.0%) unknown/uncoded cases; due to the fact that diagnoses are coded without time variable, the current coronary status at admission cannot be confirmed. PAD for peripheral arterial disease. The Charlson comorbidity index was obtained using the Stata plugin “charlson icd_sidediagnoses, index(10)”. 0: 0 points. 1: 1–2 points. 2: 3–4 points. 3: >4 points.

Additional characteristics in Supplementary Table S2.

a Numbers are median (IQR).

### The fraction of patients with in-hospital time to angiography ≤60 min and the fraction of patients with transport to hospital plus in-hospital time to angiography ≤120 min (primary outcome measure)

**Fig. 1:**
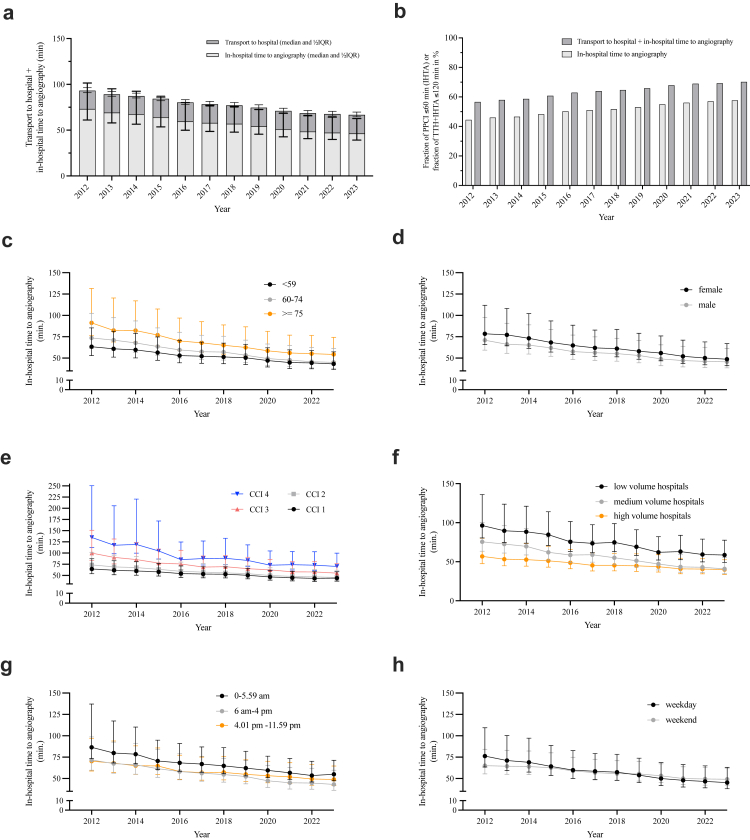
**Time to coronary angiography over time.** Values are median ± ½ interquartile range, IQR except in **b**; values in **b** are %. CCI for Charlson comorbidity index. The Charlson comorbidity index was obtained using the Stata plugin “charlson icd_sidediagnoses, index (10)”. 0: 0 points. 1: 1–2 points. 2: 3–4 points. 3: >4 points. **a** Overall time by fractions of transport to hospital (TTH) and in-hospital time to angiography (IHTA) per year (in mean ± standard deviation in Supplementary Figure S3). **b** Fraction of patients with IHTA ≤60 min or fraction of TTH + IHTA ≤120 min in %. **c** IHTA per year by patient age at admission. **d** By coded sex. **e** IHTA per year by coded Charlson comorbidity index. **f** By hospital volume based on volume tertiles. **g** Admission by time of admission, as shown. **h** Admission by weekday or weekend.

The median in-hospital time to angiography (IHTA) was 57.5 min (IQR 20.3–138.9 min) with an average of 348.7 min (standard deviation, SD, 1692.2). The median total delay (TTH + IHTA) was 84.4 min (IQR 46.2–165.9 min). The proportion of patients with IHTA ≤60 min increased from 44.5% in 2012 to 57.7% in 2023. The patients with TTH + IHTA ≤120 min increased from 56.6% in 2012 to 70.2% in 2023 (Table 1, Fig. 1, Supplementary Figure S3).

### In-hospital time to angiography: patient factors and medical system factors (secondary outcome measures)

IHTA decreased from median 73.1 min (IQR 25.2–186.6, mean 493.4 min, SD 2094.7) in 2012 to median 46.4 min (IQR 17.5–111.6) in 2023. The TTH time remained constant with a median of 11.4–11.9 min 2012–2023 (Fig. 1 a and Supplementary Figure S3). IHTA increased with age and frailty. IHTA was longer for female patients (2012: median 78.5 vs 70.9 min, respectively); this discrepancy decreased over time (2023: median 48.7 vs 45.6 min, respectively) (Fig. 1 d). Admissions between 0.00 am and 5.59 am were associated with a longer IHTA (vs 6 am–4 pm and 4.01 pm–11.59 pm) (Fig. 1 g). Admissions on a weekday had a longer IHTA 2012–2014 with a subsequent switch to longer IHTA on weekends (2023: median 49.2 min on weekends vs 45.3 min on weekdays) (Fig. 1 h). Rural hospitals had longer IHTA in 2012; this discrepancy diminished over time. Patients from a rural area code had a longer IHTA (Supplementary Figure S3g–h). Hospital properties that were found to be associated with shorter IHTA were increasing patient caseload (by annual caseload), availability of cardiac surgery, and high patient volume by hospital volume tertiles over time (Fig. 1f and Supplementary Figure S3).

### Jumper status (post-hoc analysis of secondary outcome measures)

**Fig. 2:**
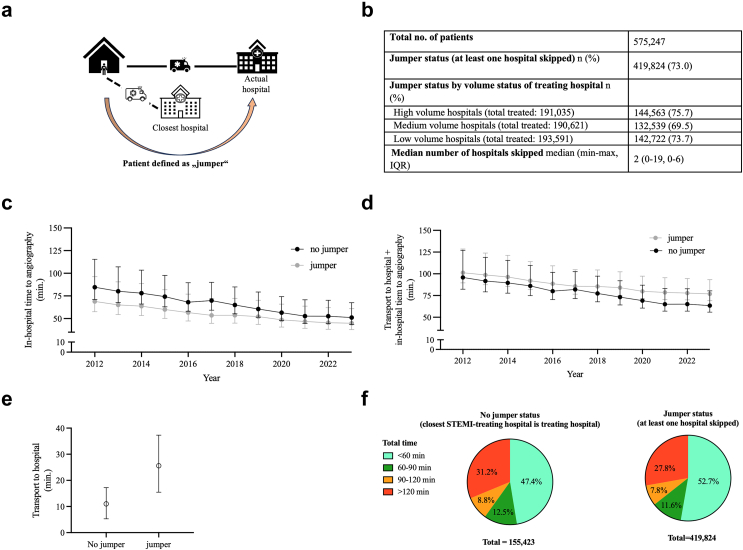
**Patient status: Jumper. a** Illustration of jumper status. A patient is considered a jumper if the treating hospital is not the closest primary percutaneous coronary intervention hospital. **b** Details on jumper status for the total cohort. **c** In-hospital time to angiography (IHTA) by jumper over time. Data are median ± ½ interquartile range, IQR. **d** Total time (transport to hospital, TTH, based on geographic routing results + IHTA) by jumper status for the total cohort. Values are median ± ½ IQR. **e** Time of TTH by jumper status. Values are median ± IQR. **f** Overall allocation of TTH + IHTA by jumper status.

Patients treated in a hospital that was not the closest STEMI treating hospital were referred to as jumpers (Fig. 2 a; Supplementary Table S3). In 419,824 cases, at least one hospital was skipped (73.0%). Jumper status was not associated with tertiles of patient volumes (73.7% vs 75.7% in low vs high volume hospitals). In jumper patients, IHTA was shorter than in non-jumper patients (2012: 68.9 min vs 84.7 min, 2023: 44.9 min vs 51.1 min) (Fig. 2 c). TTH + IHTA, in contrast, was longer in jumper patients than in non-jumper patients (2012: 101.2 min vs 95.9 min, 2023: 76.8 min vs 63.5 min) (Fig. 2 d) due to longer TTH in jumper patients (Fig. 2 e). The fraction of patients with TTH + IHTA ≤60 min was larger in jumper patients (221,438, 52.7%) in comparison to non-jumper patients (73,674, 47.4%) (Fig. 2 f). TTH intervals were heterogeneous among regions; additional TTH due to hospital skipping ranged between 1.26 min and 18.21 min by state (Supplementary Figure S4). The median number of hospitals skipped differed largely among states with a median of 9 and 11 in the metropolitan areas of Hamburg and Berlin, respectively, compared to 1 in states with larger rural areas such as Schleswig–Holstein, Rheinland-Pfalz and Mecklenburg-Vorpommern with a lower density of STEMI-treating hospitals).

### Performer status (post-hoc analysis of secondary outcome measures)

**Fig. 3:**
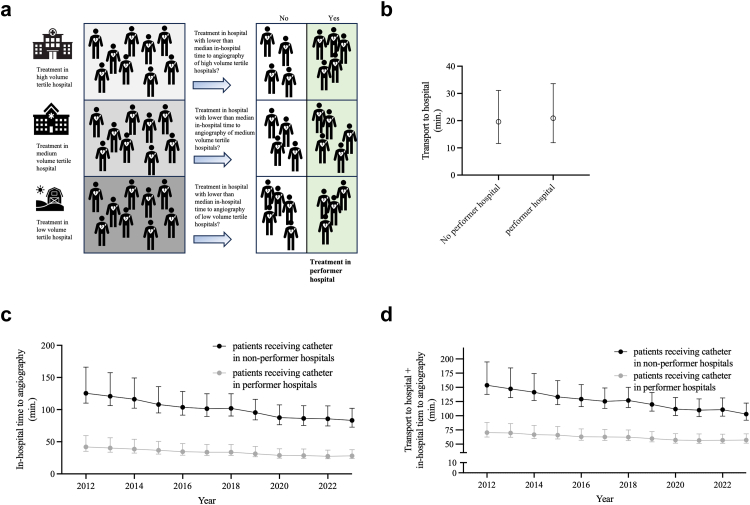
**Hospital status: Performer. a** Illustration of hospital tertiles and of performer status. All cases were assigned to hospital tertiles treating an equal number of cases in the time window of this study, resulting in an assignment to a hospital tertile for each hospital (low, medium, high volume) using the hospital identifier and the caseload of the treating hospital in an ascending order. An individual hospital is considered a performer hospital if its median in-hospital time to angiography (IHTA) is equal or lower than the median IHTA of its respective tertile. **b** Time of transport to hospital (TTH) by performer status. Values are median ± interquartile range, IQR. **c** IHTA by performer status over time. Data are median ± ½ IQR. **d** Total time (TTH based on geographic routing results + IHTA) by performer status for the total cohort. Values are median ± ½ IQR.

Performer status was defined as a shorter median IHTA than 50% of hospitals in its respective volume tertile (Fig. 3 A). 331,424 patients (57.6%) were treated in a performer hospital with a clear stratification of IHTA in longer time intervals in non-performer hospitals than in performer hospitals (2012: median 125.4 min vs 42.0 min, 2023: 83.2 min vs 28.0 min). The TTH interval was similar (Fig. 3b and c), resulting in a decreased total delay in performer hospitals (2023: median 102.9 min vs 57.3 min) (Fig. 3 d).

### Delay in time to angiography (primary outcome measures)

**Fig. 4:**
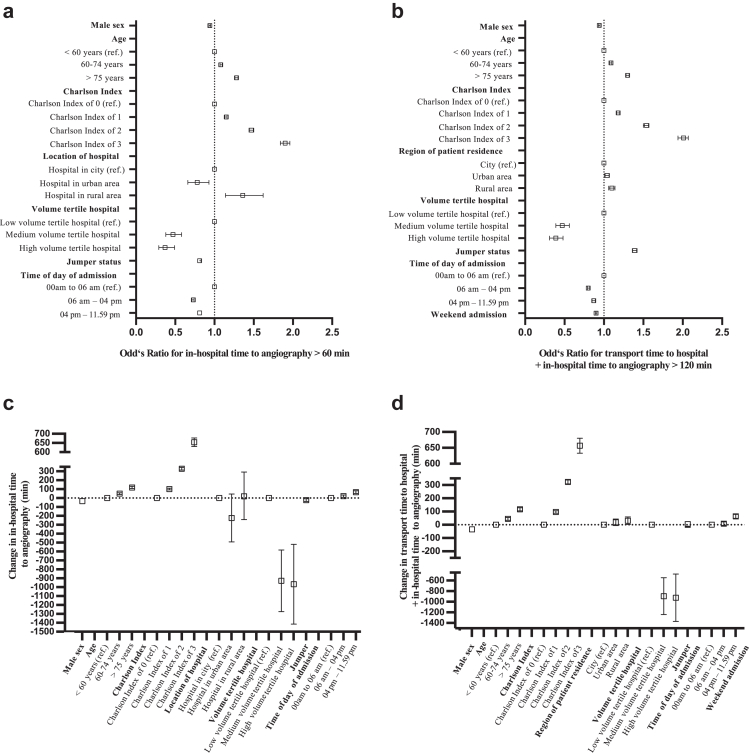
**Results from a multivariable logistic regression model for delay to angiography and from a linear regression model for change in time to angiography. a** Odd's Ratios for in-hospital time to angiography (IHTA) > 60 min. **b** Odd's Ratios for transport time to hospital + IHTA >120 min. **c** Change in IHTA in min by patient and medical system factors. The overall coefficient in min was 282.6, 95% Confidence Interval 262.8–302.5. **d** Change in transport time to hospital + IHTA in min by patient and medical system factors. The overall coefficient in min was 350.1, 95% Confidence Interval 329.9–370.3. For **b** and **d**, transferred patients were excluded. Details for this sub-cohort in Supplementary Table S3. Results in detail in Supplementary Table S4 and Supplementary Table S5.

Patient and medical system factors affecting IHTA and TTH + IHTA were analyzed as to their risk of delaying these time intervals to longer than 60 min and 120 min as primary outcome measures, respectively (transferred patients were excluded for the analysis regarding 120 min, cohort in Supplementary Table S3). In a multivariable logistic regression model, risk factors for IHTA >60 min were high comorbidity indices (Charlson comorbidity index >4 points: Odd's Ratio, OR, 1.90, 95% Confidence Interval, CI 1.84–1.96, p<.001) and increased age (>75 years: OR 1.28, CI 1.26–1.30, p<.001). Factors associated with reduced risk of exceeding this time interval were male sex (OR 0.94, CI 0.93–0.95, p<.001), high patient volume (high volume hospitals in reference to low volume hospitals: OR 0.37, CI 0.29–0.49, p<.001), jumper status (OR 0.81, CI 0.80–0.83, p<.001), and time of admission other than between 00.00 am and 5.59 am (Fig. 4 a).

Risk factors for TTH + IHTA >120 min were high comorbidity indices (Charlson comorbidity index >4 points: OR 2.01, CI 1.94–2.07, p<.001) and increased age (>75 years: OR 1.30, CI 1.28–1.32, p<.001), urban and rural residence regions (OR 1.04 and 1.10, respectively), and jumper status (OR 1.39, CI 1.36–1.42, p<.001); Factors associated with reduced risk of exceeding this time interval were male sex, high hospital volume, time of admission other than between 00.00 am and 5.59 am, and weekend admissions (OR 0.60, 0.51–0.70, p<.001) (Fig. 4b, Supplementary Figure S6, Supplementary Figure S7).

To quantify the impact of these factors, multivariable linear regression models were built. Both time intervals were prolonged the longest in case of Charlson comorbidity indices >2. Male sex was associated with a reduction of IHTA of 34.2 min (CI 24.4–44.1) and a reduction of TTH + IHTA of 34.4 min (CI 24.5–44.4) compared to women. The factor associated with the longest reduction was procedure performance in a high volume hospital (Fig. 4c and d, Supplementary Table S5).

### In-hospital mortality (primary outcome measure)

**Fig. 5:**
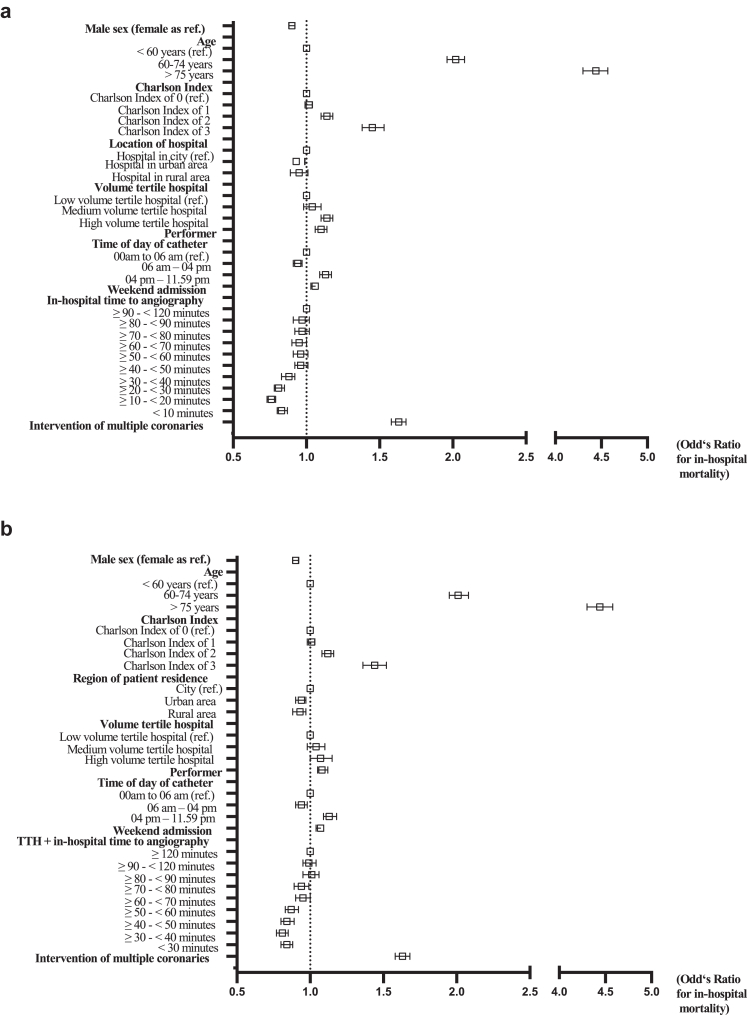
**Results from a multivariable logistic regression model for in-hospital mortality. TTH for transport to hospital, due to readability.** Coronaries short for coronary arteries. **a** In-hospital mortality as dependent variable for in-hospital time to angiography (IHTA). **b** In-hospital mortality as dependent variable for transport time to hospital + IHTA. Patient records with IHTA >120 min were pre-defined to be excluded for this analysis. For **b**, transferred patients were excluded. Details for these sub-cohorts in Supplementary Table S6 (for **a**) and in Supplementary Table S7 (for **b**). Results in detail in Supplementary Table S8.

Total in-hospital mortality was 9.5% (54,640 patients) and increased over time (8.8% in 2012, 10.4% in 2021, 10.1% in 2023) (Supplementary Table S2 and Supplementary Figure S5). 28.7% (165,205 patients) had a time to angiography longer than 120 min, with an in-hospital mortality rate of 10.0% (16,486 patients). 10.7% (61,425 patients) had a time to angiography between 10 and 20 min, with an in-hospital mortality rate of 8.4% (5129 patients) (Supplementary Table S2). In a multivariable logistic regression model (patient records with IHTA >120 min were pre-defined to be excluded for this analysis, cohorts in Supplementary Table S6 and Supplementary Table S7), risk factors for in-hospital mortality regarding in-hospital delay (one covariate being IHTA) were comorbidity (Charlson comorbidity index >4 points: OR 1.45, CI 1.38–1.53, p<.001), increased age (>75 years: OR 4.44, CI 4.30–4.57, p<.001), high-volume hospitals (OR 1.07, CI 1.00–1.14, p = 0.043), performer status (OR 1.10, CI 1.06–1.14, p<.001), intervention of multiple coronary arteries (OR 1.63, CI 1.58–1.68, p<.001), admission between 4 pm and 11.59 pm (OR 1.06, CI 1.03–1.08, p<.001), and weekend admissions (OR 1.06, CI 1.03–1.08, p<.001). Factors associated with a reduced risk of death were male sex (OR 0.90, CI 0.88–0.92, p<.001), urban hospitals (OR 0.93, CI 0.88–0.99, p = 0.023), daytime admissions (6 am–4 pm, OR 0.94, CI 0.91–0.97, p<.001), and IHTA <40 min (OR for 10–20 min: 0.76, CI 0.73–0.79, p<.001, 90–120 min as reference) (Fig. 5 a, Supplementary Table S8). A multivariable logistic regression model for in-hospital mortality regarding overall delay (one covariate being TTH + IHTA) revealed increased age, comorbidity, high volume hospitals, performer status, admission from 4 pm to 11.59 pm, weekend admissions, and intervention of multiple coronary arteries as risk factors. Factors associated with reduced risk of death were male sex, urban and rural patient residence (OR 0.94 and 0.93, respectively), daytime admissions, and TTH + IHTA <80 min (>120 min as reference, excluding patients IHTA >120 min; OR for 30–40 min: 0.81, CI 0.77–0.85, p<.001) (Fig. 5b, Supplementary Table S8, Supplementary Figure S6, Supplementary Figure S7).

In light of an increase in in-hospital mortality over time, a post-hoc analysis was performed, including year of admission into the multivariable logistic regression model. In this model, a trend toward increasing in-hospital mortality was observed as of 2019 (OR 1.06, CI 1.01–1.12, p = 0.028, in reference to 2012). A margins analysis of this regression model demonstrated a decrease of in-hospital mortality from 2012 through 2015 (8.83% in 2012, 8.50% in 2015), and a following increase (8.58% in 2016, 9.84% in 2021, 9.53% in 2023). This was observed across all age strata of this analysis (Supplementary Figure S8).

### Re-allocation of patients: results from a hospital-volume based simulation (post-hoc analysis of primary outcome measures regarding in-hospital delay and overall delay)

**Fig. 6:**
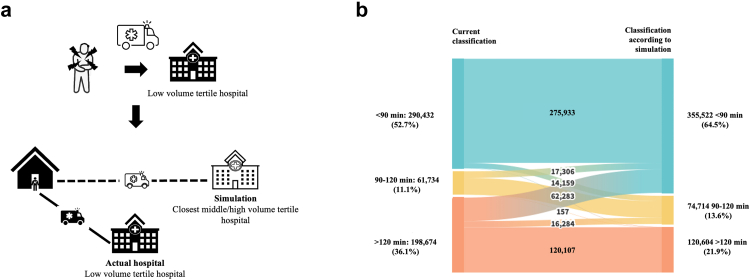
**Simulation: Re-allocation of patients from low-volume hospitals to closest medium and high-volume hospitals. a** Simulation in detail: In case a patient is transported to an individual low-volume hospital, the simulation re-allocates the patient to the closest medium- or high-volume hospital assuming a median in-hospital time to angiography in that hospital of the respective annual hospitals in the corresponding tertile. **b** Results of the re-allocation in detail using a Sankey diagram.

A simulation of re-allocation was conducted to analyze its impact on time interval distribution to TTH + IHTA <90 min, 90–120 min, or >120 min. In case a patient was transported to a low-volume hospital, the simulation re-allocated the patient to the closest medium- or high-volume hospital assuming a median IHTA of hospitals in the respective years in the corresponding tertile and applying the actual changes in transport time using geographic routing (Fig. 6a). Results of the re-allocation in detail is depicted using a Sankey diagram. Re-allocation would change the classification in 110,189 cases (19.2%), of which 62,283 (10.8% of total cohort) switch from >120 min to <90 min (Fig. 6b).

## Discussion

This is the first analysis of real-world population-based administrative data combined with geographic routing in acute ST-segment elevation myocardial infarction (STEMI) in Germany. The goal of this benchmarking approach was to identify factors contributing to delays in care for this high-incidence, time-critical condition and to examine their impact on in-hospital mortality.

We observed a reduction in in-hospital time to angiography (IHTA), with the median time decreasing from 73 min in 2012 to 46 min in 2023. In contrast, the time for transport to hospital (TTH) remained largely unchanged over the past decade. The proportion of patients achieving IHTA within 60 min increased from 44.5% in 2012 to 57.7% in 2023. Additionally, the percentage of patients meeting the combined benchmark of TTH plus IHTA ≤120 min rose from 56.6% to 70.2%, aligning with the recommended time frames outlined in the 2023 ESC guidelines.^12^ When the recommended time threshold of 120 min–approximated by the combined duration of TTH and IHTA–was exceeded, crude in-hospital mortality was 9.8%. This rate was 0.5 percentage points lower (9.3%) if the threshold was met. Similarly, patients who underwent angiography within 60 min (IHTA ≤60 min) had a mortality rate of 9.1%, compared to 9.9% in those with longer delays (>60 min). Multivariate analysis revealed that an IHTA of less than 40 min and IHTA + TTH <80 min were independently associated with a reduced risk of in-hospital death.

One important factor associated with in-hospital delay in this analysis was female sex. There is evidence of a persisting sex inequality in the care of female STEMI patients.^21^^,^^25^^,^^26^ The population attributable fraction of deaths due to not meeting the IHTA <60 min target among females was 3.7%^27^^,^^28^–corresponding to an estimated attributable 387 female deaths due to delayed PPCI in STEMI in Germany between 2012 and 2023. Several factors may contribute to this delay, including longer delays from symptom onset to first medical contact, atypical symptom presentation, and under-recognition of cardiovascular risk in women. Our findings underscore the urgent need to raise awareness of sex-specific differences in STEMI presentation, comorbidity profiles, and clinical management. Targeted strategies to reduce treatment delays in women are essential and may include public education campaigns, enhanced training for first responders, and systematic auditing of time-to-treatment metrics stratified by sex.

Other factors associated with in-hospital delay and increased mortality in this analysis included increased age, higher comorbidity burden, and presentation outside regular hours. Age is a known risk-factor of short- and long-term mortality in STEMI.^29^ Also, the study is consistent with the literature in showing worse outcomes in patients with acute myocardial infarction presenting during off-hours.^30^ This modifiable factor highlights the importance of 24/7 availability of PPCI in regional STEMI networks and the awareness of increased risk of delay in STEMI care in case of understaffing. While the findings above are in line with results from this study, it is notable that weekend presentation was associated with reduced risk of total delay in this analysis. Several factors may contribute to this finding, for example competing elective cases or resource constraints. It is to discuss whether dedicated daytime acute care catheter labs could feasibly reduce in-hospital delays, assuming adequate staffing and infrastructure. In addition, multivessel disease is known to be a risk-factor of mortality, which was confirmed in this analysis.^31^

There is evidence of an impact of prehospital triage, particularly field triage performed by ambulance services, can reduce in-hospital delays and shorten time to revascularization.^32^ In the current analysis, data on prehospital Emergency Medical Service (EMS) such as time from emergency call to EMS arrival and response times were not available. Therefore, these quality indicators within the STEMI treatment chain could not be taken into account. This study identified that hospital skipping was associated with a reduction of IHTA, concurrently resulting in an overall increase in combined TTH + IHTA. This observation may support the recommendation of heading to the closest PPCI hospital in case of pre-clinical STEMI diagnosis. However, such recommendations must be considered within the context of an established STEMI network. The impact of hospital skipping on TTH varied by region, depending on local PPCI hospital density. The balance between reducing time delays and referral to the most adequately equipped hospital in accordance with the patient's condition is an important challenge in the complexity of triage decisions. These findings highlight the logistical challenges of ensuring timely STEMI care in rural areas, where emergency medical services and PCI center availability are often limited,^33^^,^^34^ possibly profiting from telemedicine-enabled diagnosis. This study found that a city hospital and a city patient residence were associated with increased risk of mortality, an observation that requires further investigation, particularly regarding the influence of comorbidity profiles.

Over the study period, in parallel with the reduction in IHTA, in-hospital STEMI mortality increased from 8.8% in 2012 to 10.4% in 2021, and slightly declined to 10.1% in 2023. This trend aligns with the plateau in STEMI mortality observed since 2010.^35^ During the same time period, initiatives to reduce door-to-balloon time have been successfully implemented,^36^ often coinciding with reductions in in-hospital mortality, with some studies suggesting a causal link.^10^^,^^11^ Other analyses have not confirmed this association.^37^ Factors associated with short IHTA in this analysis were only partly associated with reduced in-hospital mortality. The increasing mortality over time in this analysis may in part be explained by confounding patient risk factors and selection bias—particularly the increasing use of PPCI in older and more comorbid patients over time, a trend also observed in this analysis. In this context, while it was not the intention of this study to analyze a potential impact of the SARS-CoV-2 pandemic, Covid-19 waves were found to be associated with increased mortality in STEMI patients in Germany^38^ and to increased IHTA.^39^ In this analysis, in a margins analysis of the multivariable logistic regression results, an increase in mortality peaked in 2021, coinciding with high incidences of SARS-CoV-2 in Germany. While this peak was observed, a trend toward increasing in-hospital mortality in STEMI patients was found starting 2019 in the current study, predating the onset of the SARS-CoV-2 pandemic in Germany. Therefore, this trend must be assumed to be multifactorial and warrants further investigations.

In contrast to a previous German study, the current analysis found an increased in-hospital mortality in high-volume hospitals.^40^ This observation is not explained by shorter IHTA in high-volume hospitals.^41^ In this analysis, simulation of patient reallocation from low-to higher-volume nearby centers reduced the proportion with TTH + IHTA >120 min by 14.2%, suggesting potential benefit in reducing total ischemic time. The implications of this simulation must be considered both in the context of total ischemic time reduction and a volume-outcome analysis. Data on stratified volume-outcome analyses in STEMI patients are scarce, while existing evidence found no volume outcome relationship^42^ or an association between higher caseload and lower mortality.^43^ Increased mortality in the high-volume STEMI centers may reflect unmeasured comorbidity or procedural complexity. Comorbidity burden was reduced in medium volume hospitals and was highest in low and high-volume hospitals. Finally, the availability of cardiac surgery was associated with reduced IHTA, but did not correlate with lower in-hospital mortality.^44^ These findings underscore the complexity of volume-outcome relationships and highlight the need for further investigation into how hospital characteristics, patient profiles, and system-level factors interact to influence STEMI outcomes.

There are several limitations to this analysis. First, no causal conclusions can be drawn from this analysis due to its retrospective nature. Second, admission times may be inaccurately coded —especially in emergencies— potentially leading to underestimated IHTA. The dataset lacked details on the route of presentation (self-admission vs emergency medical services), potentially overestimating time to angiography in self-presenters. The available information on location was restricted to area codes; the geographic routing results must be interpreted in light of underlying assumptions, i.e. travelling from the center of the patient's area code to the center of the area code of the treating hospital using the most direct route. Data on prehospital emergency medical service and place of symptom onset was not available. Clinical data beyond patient characteristics and coded diagnoses codes (ICD) and procedural coding (OPS) were not available, most notably on time from symptom onset to treatment, hypotension at admission, details regarding the PPCI beyond coding, cardiac ultrasound and heart function, out-of-hospital cardiac arrest, or smoking status, introducing residual confounding, which has to be considered in the interpretation of all findings. Some of these factors, like acute heart failure at admission, may introduce bias toward increased mortality and increased IHTA due to prior stabilization measures.^45^ For the outcome measure in-hospital mortality, due to the possibility of patients being late presenters,^12^ patient records with IHTA >120 min were excluded, while this patient cohort benefits from PCI treatment.^46^ The analysis is restricted to in-hospital data. Therefore, no data is available regarding readmission rates, with 30-day readmission rates of approximately 12%, of which approximately 50% were for non-cardiac reasons,^47^ and long-term outcomes, i.e. heart function at follow-up visits. In addition, the generalizability of these results beyond Germany, i.e. to different health-care systems and patient cohorts with differing age, comorbidity and social structures, is uncertain.^48^ Information on social status and race are not included in German in-hospital data.^49^

In conclusion, this large, real-world analysis of in-hospital data combined with a geographic routing approach in STEMI patients in Germany revealed a significant decrease in in-hospital time to angiography between 2012 and 2023. The integration of geographic routing-based surrogates for transport time to hospital provides comprehensive benchmarking of pre- and in-hospital delays in STEMI care. Risk factor analyses for prolonged IHTA (>60 min), extended total ischemic time (TTH + IHTA >120 min), and in-hospital mortality underscored critical areas for improvement. These include enhancing care during out-of-hours presentations and ensuring equitable compliance with guideline-recommended time targets across sexes. Additionally, elderly and comorbid patients were found to be at increased risk for both treatment delays and adverse outcomes, highlighting the need for targeted strategies to optimize STEMI care in these vulnerable populations.

## Contributors

PES was responsible for data curation, formal analysis, literature search, resources, validation, visualisation, writing of the original draft, reviewing and editing, and data access and verification. UL was responsible for and/or involved in resources, validation, visualisation, writing of the original draft, reviewing and editing, supervision, and project administration. PB was part of methodology, investigation, data curation, formal analysis, literature search, resources, validation, visualisation, and writing of the original draft. JD was involved in methodology, investigation, data curation, formal analysis, literature search, reviewing and editing the manuscript editing, software, and supervision. AW contributed to the aspects of methodology, investigation, resources, validation, reviewing and editing, data access and verification, supervision, and project administration. KU conceptualized the project, and was responsible for methodology, investigation, data curation, formal analysis, literature search, resources, validation, visualisation, writing, reviewing and editing, data access and verification, supervision, and project administration.

All authors made the decision to submit the manuscript for publication. All authors meet the International Committee of Medical Journal Editors criteria for authorship for this article and have given their approval for this version to be published.

## Data sharing statement

Since all data accessing and analysis steps were conducted in collaboration with the Federal Statistical Office in Germany, Gustav-Stresemann-Ring 11, 65,189 Wiesbaden, represented by their president, Dr. Ruth Brand, and since all data are protected by their guidelines regarding highly sensitive patient-record data, no individual data can be made available. However, in accordance with their guidelines, all data can be permanently accessed after acceptance of an application for data usage by the Federal Statistical Office. Using the inclusion criteria, the same patient cohort as used for this analysis can be identified. It is possible to contact the corresponding author of this Study, Dr. Konstantin Uttinger, to request access to the Stata protocol for a detailed analysis of the coding steps for identification of the study cohort.

## Editor note

The Lancet Group takes a neutral position with respect to territorial claims in published maps and institutional affiliations.

## Declaration of interests

There are no DOIs associated with this study, but the authors have received the following honoraria etc. within the past 3 years: PES has received honoraria from Daiichi Sankyo, Amgen, Novartis, and Synlab, and from Novartis for an advisory board. UL has received honoraria from Amgen, AstraZeneca, Bayer, Boehringer, Daiichi-Sankyo, Lilly, MSD, Novartis, NovoNordisk, Pfizer, Sanofi, Synlab, and from Amgen, Boehringer, Daiichi-Sankyo, MSD, Novartis, and Sanofi for an advisory board, and is a member of the board of Deutsche Gesellschaft für Kardiologie, the German Society of Cardiology. KU was supported by MSNZ Frankfurt in the scope of a postdoctoral fellowship. PB, JD, and AW have nothing to declare.

## Appendix A Supplementary data

Supplementary Figures and Tables

Translated Abstract
