# Supplementary material for: Time to coronary angiography and revascularization in 575,247 patients with STEMI from 2012 to 2023: a retrospective population-based cohort study
Source: Lancet Reg Health Eur. 2025 Dec 29;62:101576. doi: 10.1016/j.lanepe.2025.101576 (PMC12803846; doi:10.1016/j.lanepe.2025.101576)
Supplement: Supplementary Figures and Tables [file mmc1.docx]

# Supplements

| **ICD Diagnoses** |
| --- |
| Main diagnosis: I21.0-I21.3 (STEMI)  Other main diagnoses for hospital status identification: I21.4, I20, I25  Coronary status: I25.11-I.25.16  Comorbidities: E66, I10, I11.9, I12.9, I13.9, I15, I13.1, I13.2, N03, N04, N05, N07, N08, N11, N12, N14, N15, N16, N18, N19, Z99.2, E10, E11, E12, E13, E14, I48, I702, I50, I11.0, I13.0, I13.2, E78.0, E78.2, E78.5 |
| **OPS Codes** |
| Left ventricular coronary angiography: 1-275  Procedures during left ventricular coronary angiography: 8-837  Lysis: 8-020.8  Bypass surgery: 5-361 |

**Supp. Table 1: ICD Diagnoses and OPS codes for patient identification.** ICD for International Statistical Classification of Diseases and Related Health Problems, Version 10, German modification. OPS for “Operationen und Prozedurenschlüssel”, a German procedural coding system. The Charlson comorbidity index was obtained using the Stata plugin “charlson icd_sidediagnoses, index(10)”.

**Supp. Figure 1: Flowchart of inclusion.** Details on patient identification using ICD and OPS (procedural code) criteria in Supp. Table 1. Transfer-status on patient level was identified using a categorical admission code (admission from other hospital with a length of stay in previous hospital < 24h). Percentages of discarded patient records refer to the previous total.

**Supp. Figure 2: Regional heterogeneities in STEMI-incidence and density of hospitals with primary percutaneous coronary intervention (PPCI) over time.** Colour per state indicates annual cases of STEMI per 100,000 inhabitants in 2012 (left) and 2023 (right). Diameter of points represent density of PPCI hospitals in the respective state in 2012 (left) and 2023 (right); for illustrative purposes, the point location was chosen to be in the capitol of the respective states.

**Supp. Figure 3: In-hospital time to angiography, age, Comorbidity index and length of stay over time.** Values are mean ± standard deviation (SD) in **a** and **b**. In **c** and **d**, values are mean ± ½ SD due to presentability. In **e**-**h**, values are median ± ½ interquartile range, IQR. P-values from Kruskal-Wallis testing were <·001 for a change in B, C and D between 2012 and 2023. Post-hoc tests demonstrated a change in terms of an increase in **b**, an increase between 2012 and 2020 in **c** with a following stability until 2023 (with an overall increase between 2012 and 2023, p<·001), and a decrease in **d**. **e** In-hospital time from admission to angiography, IHTA, by availability of cardiac surgery; for identification of this status on hospital level, bypass-surgery cases were initially identified and then excluded from further analyses, Supp. Figure 1. **f** By hospital volume of treating hospital; patients treated per year identified using a constant hospital identifier. **g** IHTA by location of treating hospital: urban, suburban, and rural, as shown. **h** IHTA by region of patient residence: urban, suburban, and rural.

|  | **Overall** |
| --- | --- |
| **Total no. of patients** | 575,247 |
| Mortality overall n (%) | 54,640 (9·5) |
| **Admission to high volume tertile hospital**  n (%) | 189,907 (33.0), number of hospitals: 83, number of annually treated patients (median, IQR): 185·8, 160·8-254; Charlson mean 1·28, SD 1·48, age mean 64·7, SD 13·0 |
| Admission to medium volume tertile hospital n (%) | 192,296 (33·4), number of hospitals: 152, number of annually treated patients (median, IQR): 110·8, 99·1-128·2; Charlson mean 1·21, SD 1·46, age mean 64·7, SD 13·0 |
| Admission to low volume tertile hospital n (%) | 193,044 (33·6), number of hospitals: 644, number of annually treated patients (median, IQR): 57·3, 39·1-72·3; Charlson mean 1·29, SD 1·52, age mean 65·5, SD 13·0 |
| **Admission to center with availability of cardiac surgery** n (%) | 127,742 (22·2) |
| **Admission to hospital coded to be in urban area** n (%) | 266,794 (46·4) |
| Admission to hospital coded to be in suburban area n (%) | 176,728 (30·7) |
| Admission to hospital coded to be in rural area n (%) | 131,725 (22·9) |
| **Patient area code: urban area** n (%) | 258,120 (44·9) |
| Patient area code: suburban area n (%) | 183,190 (31·9) |
| Patient area code: ruralurban area n (%) | 133,937 (23·3) |
| **In-hospital time to angiography** n (%) |  |
| < 10 min | 80,738 (14·0) |
| Mortality n (%) | 7,295 (9·0) |
| ≥ 10 - < 20 min | 61,425 (10·7) |
| Mortality n (%) | 5,129 (8·4) |
| ≥ 20 - < 30 min | 47,795 (8·3) |
| Mortality n (%) | 4,202 (8·8) |
| ≥ 30 - < 40 min | 39,946 (6·9) |
| Mortality n (%) | 3,722 (9·3) |
| ≥ 40 - < 50 min | 34,604 (6·0) |
| Mortality n (%) | 3,461 (10·0) |
| ≥ 50 - <60 min | 30,606 (5·3) |
| Mortality n (%) | 3,034 (9·9) |
| ≥ 60 - < 70 min | 26,319 (4·6) |
| Mortality n (%) | 2,562 (9·7) |
| ≥ 70 - < 80 min | 22,500 (3·9) |
| Mortality n (%) | 2,198 (9·8) |
| ≥ 80 - < 90 min | 19,508 (3·4) |
| Mortality n (%) | 1,894 (9·7) |
| ≥ 90 - ≤ 120 min | 46,601 (8·1) |
| Mortality n (%) | 4,657 (10·0) |
| > 120 min | 165,205 (28·7) |
| Mortality n (%) | 16,486 (10·0) |

**Supp. Table 2: Patient characteristics, in-hospital time to angiography and in-hospital mortality in detail in the total cohort.** Numbers in parantheses are %.

| **Total no. of patients** | 550,840 |
| --- | --- |
| **Age** (years) * | 64 (55-75) |
| ≤ 59 n (%) | 202,162 (36·7) |
| 60-74 n (%) | 200,861 (36·5) |
| > 75 n (%) | 147,817 (26·8) |
| **No. of females** n (%) | 156,419 (28·4) |
| **Sex ratio** (M:F) | 2·52 |
| **Charlson comorbidity index** n (%) |  |
| 0 | 205,357 (37·3) |
| 1 | 256,242 (46·5) |
| 2 | 67,087 (12·2) |
| 3 | 22,154 (4·0) |
| **Time of admission** n (%) |  |
| 0.00 am – 5.59 am | 70,680 (12·9) |
| 6.00 am – 4.59 pm | 340,412 (61·8) |
| 5 pm – 11.59 pm | 139,568 (25·3) |
| **Weekend admission** n (%) | 131,015 (23·8) |
| **Length of stay** (days)* | 6 (4-8) |
| **Mortality overall** n (%) | 52,270 (9.5) |
| **In-hospital time to angiography** (min) median (IQR), mean (SD) | 57·9 (20·6-139·0), 347·4 (1,674·1) |
| **In-hospital time to angiography** n (%) |  |
| < 10 min | 76,240 (13·8) |
| Mortality n (%) | 6,892 (9·0) |
| ≥ 10 - < 20 min | 58,354 (10·6) |
| Mortality n (%) | 4,829 (8·3) |
| ≥ 20 - < 30 min | 45,877 (8·3) |
| Mortality n (%) | 4,022 (8·8) |
| ≥ 30 - < 40 min | 38,413 (7·0) |
| Mortality n (%) | 3,575 (9·3) |
| ≥ 40 - < 50 min | 33,306 (6·1) |
| Mortality n (%) | 3,358 (10·1) |
| ≥ 50 - <60 min | 29,484 (5·4) |
| Mortality n (%) | 2,931 (9·9) |
| ≥ 60 - < 70 min | 25,356 (4·6) |
| Mortality n (%) | 2,464 (9·7) |
| ≥ 70 - < 80 min | 21,675 (3·9) |
| Mortality n (%) | 2,119 (9·8) |
| ≥ 80 - < 90 min | 18,764 (3·4) |
| Mortality n (%) | 1,822 (9·7) |
| ≥ 90 - ≤ 120 min | 44,867 (8·2) |
| Mortality n (%) | 4,472 (10·0) |
| > 120 min | 158,504 (28·8) |
| Mortality n (%) | 15,786 (10·0) |
| **Overall time: Transport to hospital + in-hospital time to angiography** (min) median (IQR), mean (SD) | 84·3 (46·1-165·7), 372·0 (1,673·9) |
| **Transport to hospital + in-hospital time to angiography > 120 min** n (%) | 198,674 (36·1) |
| Mortality n (%) | 19,491 (9·8) |
| **Transport to hospital + in-hospital time to angiography 90-120 min** n (%) | 61,734 (11·2) |
| Mortality n (%) | 5,989 (9·7) |
| **Transport to hospital + in-hospital time to angiography <90 min** n (%) | 290,432 (52·7) |
| Mortality n (%) | 26,790 (9·2) |
| **Transport to hospital + in-hospital time to angiography** n (%) |  |
| ≥120 min | 198,673 (36·1) |
| Mortality n (%) | 19,491 (9·8) |
| ≥90 - <120 min | 61,734 (11·2) |
| Mortality n (%) | 5,989 (9·7) |
| ≥80 - <90 min | 27,297 (5·0) |
| Mortality n (%) | 2,699 (9·9) |
| ≥70 - <80 min | 31,794 (5·8) |
| Mortality n (%) | 3,018 (9·5) |
| ≥60 - <70 min | 36,119 (6·6) |
| Mortality n (%) | 3,510 (9·7) |
| ≥50 - <60 min | 40,869 (7·4) |
| Mortality n (%) | 3,691 (9·0) |
| ≥40 - <50 min | 43,402 (7·9) |
| Mortality n (%) | 3,851 (8·9) |
| ≥30 - <40 min | 43,484 (7·9) |
| Mortality n (%) | 3,748 (8·6) |
| <30 min | 67,468 (12·2) |
| Mortality n (%) | 6,273 (9·3) |

**Supp. Table 3: Study population and patient characteristics of total cohort after dropping admissions from other hospitals.** Values in parentheses are percentages of total in the patient group unless otherwise indicated. The Charlson comorbidity index was obtained using the Stata plugin “charlson icd_sidediagnoses, index(10)”. 0: 0 points. 1: 1-2 points. 2: 3-4 points. 3: >4 points. * Numbers are median (interquartile range, IQR). SD for standard deviation.


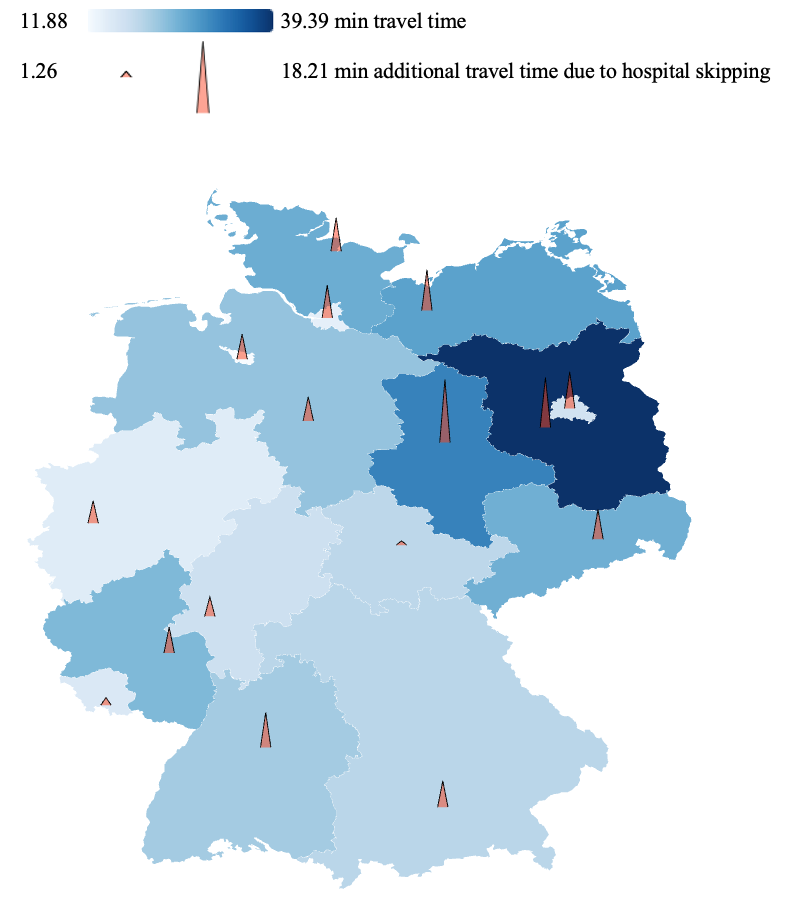


**Supp. Figure 4: Regional heterogeneities in transport to hospital: results of the geographic routing by states.** Colours by state represent median transport to hospital (TTH) in min. Spikes represent additional TTH due to hospital skipping (time difference in min between time from patient location to closest primary percutaneous coronary intervention hospital and time from patient to treating hospital in min) in the respective state; for illustrative purposes, the point location was chosen to be in the capitol of the respective states.

|  |  | **Multivariable logistic regression OR for in-hospital time > 60 min** (“In-hospital time to angiography”) **or total time > 120 min** (“Transport to hospital + in-hospital time to angiography”) | |
| --- | --- | --- | --- |
|  |  | Odd’s Ratio [95% CI] | P-value |
| In-hospital time to angiography | **Age (years)** |  |  |
|  | ≤ 59 | 1 |  |
|  | 60–74 | 1·08 [1·07-1·10] | <·001 |
|  | ≥ 75 | 1·28 [1·26-1·30] | <·001 |
|  | **Sex** |  |  |
|  | female | 1 |  |
|  | male | 0·94 [0·93-0·95] | <·001 |
|  | **Charlson comorbidity index** |  |  |
|  | 0 | 1 |  |
|  | 1 | 1·15 [1·13-1·16] | <·001 |
|  | 2 | 1·47 [1·44-1·50] | <·001 |
|  | 3 | 1·90 [1·84-1·96] | <·001 |
|  | **Location of hospital** |  |  |
|  | Hospital in city | 1 |  |
|  | Hospital in urban area | 0·78 [0·66-0·93] | 0·005 |
|  | Hospital in rural area | 1·36 [1·14-1·62] | 0·001 |
|  | **Volume tertile hospital** |  |  |
|  | Low volume tertile hospital | 1 |  |
|  | Medium volume tertile hospital | 0·47 [0·38-0·58] | <·001 |
|  | High volume tertile hospital | 0·37 [0·29-0·49] | <·001 |
|  | **Jumper** | 0·81 [0·80-0·83] | <·001 |
|  | **Time of day of admission** |  |  |
|  | 0.00 am – 5.59 am (ref.) | 1 |  |
|  | 6.00 am – 4.59 pm | 0·73 [0·71-0·74] | <·001 |
|  | 5 pm – 11.59 pm | 0·81 [0·79-0·82] | <·001 |
| Transport to hospital + in-hospital time to angiography | **Age (years)** |  |  |
|  | ≤ 59 | 1 |  |
|  | 60–74 | 1·09 [1·07-1·10] | <·001 |
|  | ≥ 75 | 1·30 [1·28-1·32] | <·001 |
|  | **Sex** |  |  |
|  | female | 1 |  |
|  | male | 0·94 [0·93-0·95] | <·001 |
|  | **Charlson comorbidity index** |  |  |
|  | 0 | 1 |  |
|  | 1 | 1·18 [1·16-1·20] | <·001 |
|  | 2 | 1·54 [1·50-1·57] | <·001 |
|  | 3 | 2·01 [1·94-2·07] | <·001 |
|  | **Region of patient residence** |  |  |
|  | City | 1 |  |
|  | Urban area | 1·04 [1·01-1·07] | 0·019 |
|  | Rural area | 1·10 [1·06-1·14] | <·001 |
|  | **Volume tertile hospital** |  |  |
|  | Low volume tertile hospital | 1 |  |
|  | Medium volume tertile hospital | 0·47 [0·39-0·56] | <·001 |
|  | High volume tertile hospital | 0·39 [0·31-0·48] | <·001 |
|  | **Jumper** | 1·39 [1·36-1·42] | <·001 |
|  | **Time of day of admission** |  |  |
|  | 0.00 am – 5.59 am (ref.) | 1 |  |
|  | 6.00 am – 4.59 pm | 0·80 [0·79-0·82] | <·001 |
|  | 5 pm – 11.59 pm | 0·87 [0·85-0·89] | <·001 |
|  | **Weekend admission** | 0·90 [0·89-0·91] | <·001 |

**Supp. Table 4: Results from a multivariable logistic regression model: Risk of in-hospital time to angiography > 60 min and risk of transport to hospital + in-hospital time to angiography > 120 min.** Multivariable results stem from a multivariable logistic regression mixed effect logistic regression model (generalized linear model: binomial, link function: logit, random effect: constant hospital identifier). Area under the curve: 0·74, 95% Confidence Interval, CI, 0·74-0·74 (In-hospital time to angiography), 0·72, CI, 0·72-0·72 (Transport to hospital + in-hospital time to angiography). The Charlson comorbidity index was obtained using the Stata plugin “charlson icd_sidediagnoses, index(10)”. 0: 0 points. 1: 1-2 points. 2: 3-4 points. 3: >4 points. Calibration plots in Supp. Figure 6, Forest plot of AUC in Supp. Figure 7. For the IHTA model, the maximum variance inflation factor was found for age groups with a variance inflation factor of 1·15. For the IHTA+TTH model, the maximum variance inflation factor was found for age groups with a variance inflation factor of 1·14.

|  |  | **Multivariable linear regression: change in in-hospital time to angiography in min** (“In-hospital time to angiography”) **or change in total time** (“Transport to hospital + in-hospital time to angiography”) | |
| --- | --- | --- | --- |
|  | **Coefficient** for multivariable results [95% CI], p-value | 1,174·28 [953·72-1,394·85] | <·001 |
|  |  | Change in min [95% CI] | P-value |
| In-hospital time to angiography | **Age (years)** |  |  |
|  | ≤ 59 (ref.) | 0 |  |
|  | 60–74 | 49·48 [39·23-59·73] | <·001 |
|  | ≥ 75 | 118·70 [106·92-130·49] | <·001 |
|  | **Sex** |  |  |
|  | female (ref.) | 0 |  |
|  | male | -34·24 [-44·08- -24·40] | <·001 |
|  | **Charlson comorbidity index** |  |  |
|  | 0 (ref.) | 0 |  |
|  | 1 | 100·25 [90·50-110·01] | <·001 |
|  | 2 | 328·79 [313·85-343·74] | <·001 |
|  | 3 | 653·91 [630·68-677·13] | <·001 |
|  | **Location of hospital** |  |  |
|  | Hospital in city (ref.) | 0 |  |
|  | Hospital in urban area | -223·84 [-492·71-45·04] | 0·103 |
|  | Hospital in rural area | 19·68 [-252·09-291·45] | 0·887 |
|  | **Volume tertile hospital** |  |  |
|  | Low volume tertile hospital (ref.) | 0 |  |
|  | Medium volume tertile hospital | -928·47 [-1,274·2- -582·74] | <·001 |
|  | High volume tertile hospital | -966·38 [-1,413·11- -519·65] | <·001 |
|  | **Jumper** |  |  |
|  | No jumper (ref.) | 0 |  |
|  | Jumper | -23·55 [-38·59- -8·50] | 0·002 |
|  | **Time of day of admission** |  |  |
|  | 0.00 am – 5.59 am (ref.) | 0 |  |
|  | 6.00 am – 4.59 pm | 21·06 [7·81-34·32] | 0·002 |
|  | 5 pm – 11.59 pm | 66·02 [51·26-80·78] | <·001 |
|  | **Coefficient** for multivariable results [95% CI], p-value | 1,129·94 [971·76-1,288·12] | <·001 |
| Transport to hospital + in-hospital time to angiography | **Age in years** |  |  |
|  | ≤ 59 (ref.) | 0 |  |
|  | 60–74 | 44·36 [34·02-54·69] | <·001 |
|  | ≥ 75 | 116·62 [104·72-128·52] | <·001 |
|  | **Sex** |  |  |
|  | female (ref.) | 0 |  |
|  | male | -34·42 [-44·36- -24·48] | <·001 |
|  | **Charlson comorbidity index** |  |  |
|  | 0 (ref.) | 0 |  |
|  | 1 | 96·17 [86·33-106·01] | <·001 |
|  | 2 | 322·79 [307·68-337·90] | <·001 |
|  | 3 | 656·54 [633·04-680·03] | <·001 |
|  | **Region of patient residence** |  |  |
|  | City (ref.) | 0 |  |
|  | Urban area | 18·24 [-6·23-42·71] | 0·144 |
|  | Rural area | 30·80 [2·68-58·91] | 0·032 |
|  | **Volume tertile hospital** |  |  |
|  | Low volume tertile hospital (ref.) | 0 |  |
|  | Medium volume tertile hospital | -894·63 [-1,241·32- -547·94] | <·001 |
|  | High volume tertile hospital | -924·37 [-1,371·31- -477·43] | <·001 |
|  | **Jumper** |  |  |
|  | No jumper (ref.) | 0 |  |
|  | Jumper | 4·49 [-10·81-19·80] | 0·565 |
|  | **Time of day of admission** |  |  |
|  | 0.00 am – 5.59 am (ref.) | 0 |  |
|  | 6.00 am – 4.59 pm | 8·45 [-4·92-21·81] | 0·216 |
|  | 5 pm – 11.59 pm | 63·59 [48·70-78·47] | <·001 |
|  | **Weekend admission** |  |  |
|  | Weekday adm. (ref.) | 0 |  |
|  | Weekend admission | -216·01 [-226·28- -205·75] | <·001 |

**Supp. Table 5: Results from a multivariable linear regression model: Change of in-hospital time to angiography and change of transport to hospital + time to angiography.** Multivariable results stem from a multivariable mixed effect linear regression model (random effect: constant hospital identifier). The Charlson comorbidity index was obtained using the Stata plugin “charlson icd_sidediagnoses, index(10)”. 0: 0 points. 1: 1-2 points. 2: 3-4 points. 3: >4 points. For the IHTA model, the maximum variance inflation factor was found for age groups with a variance inflation factor of 1·15. For the IHTA+TTH model, the maximum variance inflation factor was found for age groups with a variance inflation factor of 1·14.

**Supp. Figure 5: Number of STEMI cases and in-hospital mortality per year.** Numbers are n (left y-axis) and % (right y-axis).

| **Total no. of patients** | 410,042 |
| --- | --- |
| **Age** (years) * | 64 (55-74) |
| ≤ 59 n (%) | 157,976 (38·5) |
| 60-74 n (%) | 149,771 (36·5) |
| > 75 n (%) | 102,295 (25·0) |
| **No. of females** n (%) | 112,660 (27·5) |
| **Sex ratio** (M:F) | 2·64 |
| **Charlson comorbidity index** n (%) |  |
| 0 | 159,705 (39·0) |
| 1 | 191,460 (46·7) |
| 2 | 45,467 (11·1) |
| 3 | 13,410 (3·3) |
| **Time of admission** n (%) |  |
| 0.00 am – 5.59 am | 50,889 (12·4) |
| 6.00 am – 4.59 pm | 253,960 (61·9) |
| 5 pm – 11.59 pm | 105,193 (25·7) |
| **Weekend admission** n (%) | 102,980 (25·1) |
| **Admission from different hospital** n (%) | 17,706 (4·3) |
| **In-hospital time to angiography** (min) median (IQR), mean (SD) | 33·6 (13·4-64·5), 41·4 (32·9) |
| **Overall time: Transport to hospital + in-hospital time to angiography** (min) median (IQR), mean (SD) | 60·4 (37·3-91·7), 66·7 (37·7) |
| **Length of stay** (days)* | 6 (4-8) |
| **Mortality overall** n (%) | 38,154 (9·3) |

**Supp. Table 6: Study population and patient characteristics of total cohort after dropping in-hospital time to angiography > 120 min.** Values in parentheses are percentages of total in the patient group unless otherwise indicated. The Charlson comorbidity index was obtained using the Stata plugin “charlson icd_sidediagnoses, index(10)”. 0: 0 points. 1: 1-2 points. 2: 3-4 points. 3: >4 points. * Numbers are median (interquartile range, IQR). SD for standard deviation.

| **Total no. of patients** | 392,336 |
| --- | --- |
| **Age** (years) * | 64 (55-74) |
| ≤ 59 n (%) | 151,348 (38·6) |
| 60-74 n (%) | 143,655 (36·6) |
| > 75 n (%) | 97,333 (24·8) |
| **No. of females** n (%) | 107,214 (27·3) |
| **Sex ratio** (M:F) | 2·66 |
| **Charlson comorbidity index** n (%) |  |
| 0 | 153,112 (39·0) |
| 1 | 183,375 (46·7) |
| 2 | 43,188 (11·0) |
| 3 | 12,661 (3·2) |
| **Time of admission** n (%) |  |
| 0.00 am – 5.59 am | 48,798 (12·4) |
| 6.00 am – 4.59 pm | 243,629 (62·1) |
| 5 pm – 11.59 pm | 99,909 (25·5) |
| **Weekend admission** n (%) | 98,002 (25·0) |
| **Length of stay** (days)* | 6 (4-8) |
| **In-hospital time to angiography** (min) median (IQR), mean (SD) | 33·9 (13·6-64-8), 41·6 (32·9) |
| **Overall time: Transport to hospital + in-hospital time to angiography** (min) median (IQR), mean (SD) | 60·2 (37·2-91·6), 66·6 (37·6) |
| **Transport to hospital + in-hospital time to angiography** n (%) |  |
| ≥120 min | 40,169 (10·2) |
| Mortality n (%) | 3,705 (9·2) |
| ≥90 - <120 min | 61,734 (15·7) |
| Mortality n (%) | 5,989 (9·7) |
| ≥80 - <90 min | 27,297 (7·0) |
| Mortality n (%) | 2,699 (9·9) |
| ≥70 - <80 min | 31,794 (8·1) |
| Mortality n (%) | 3,018 (9·7) |
| ≥60 - <70 min | 36,119 (9·2) |
| Mortality n (%) | 3,510 (9·7) |
| ≥50 - <60 min | 40,869 (10·4) |
| Mortality n (%) | 3,691 (9·0) |
| ≥40 - <50 min | 43,402 (11·1) |
| Mortality n (%) | 3,851 (8·9) |
| ≥30 - <40 min | 43,484 (11·1) |
| Mortality n (%) | 3,748 (8·6) |
| <30 min | 67,468 (17·2) |
| Mortality n (%) | 6,273 (9·3) |

**Supp. Table 7: Study population and patient characteristics of total cohort after dropping admissions from other hospitals and in-hospital time to angiography > 120 min.** Values in parentheses are percentages of total in the patient group unless otherwise indicated. The Charlson comorbidity index was obtained using the Stata plugin “charlson icd_sidediagnoses, index(10)”. 0: 0 points. 1: 1-2 points. 2: 3-4 points. 3: >4 points. *Numbers are median (IQR).

|  |  | **Multivariable logistic regression model for in-hospital mortality** | |
| --- | --- | --- | --- |
|  |  | Odd’s Ratio [95% CI] | P-value |
| In-hospital time to angiography | **Age (years)** |  |  |
|  | ≤ 59 | 1 |  |
|  | 60–74 | 2·02 [1·96-2·08] | <·001 |
|  | ≥ 75 | 4·44 [4·30-4·57] | <·001 |
|  | **Sex** |  |  |
|  | female | 1 |  |
|  | male | 0·90 [0·88-0·92] | <·001 |
|  | **Charlson comorbidity index** |  |  |
|  | 0 | 1 |  |
|  | 1 | 1·02 [0·99-1·04] | ·156 |
|  | 2 | 1·14 [1·10-1·18] | <·001 |
|  | 3 | 1·45 [1·38-1·53] | <·001 |
|  | **Location of hospital** |  |  |
|  | Hospital in city | 1 |  |
|  | Hospital in urban area | 0·93 [0·88-0·99] | 0·023 |
|  | Hospital in rural area | 0·95 [0·89-1·01] | 0·093 |
|  | **Volume tertile hospital** |  |  |
|  | Low volume tertile hospital | 1 |  |
|  | Medium volume tertile hospital | 1·04 [0·98-1·10] | 0·181 |
|  | High volume tertile hospital | 1·07 [1·00-1·15] | 0·043 |
|  | **Performer** | 1·10 [1·06-1·14] | <.001 |
|  | **Time of day of admission** |  |  |
|  | 0.00 am – 5.59 am (ref.) | 1 |  |
|  | 6.00 am – 4.59 pm | 0·94 [0·91-0·97] | 0·001 |
|  | 5 pm – 11.59 pm | 1·13 [1·09-1·17] | <·001 |
|  | **Weekend admission** | 1·06 [1·03-1·08] | <·001 |
|  | **In-hospital time to angiography** |  |  |
|  | ≥90 - <120 min | 1 |  |
|  | ≥80 - <90 min | 0·97 [0·91-1·02] | 0·243 |
|  | ≥70 - <80 min | 0·97 [0·92-1·02] | 0·249 |
|  | ≥60 - <70 min | 0·95 [0·90-1·00] | 0·076 |
|  | ≥50 - <60 min | 0·96 [0·91-1·01] | 0·107 |
|  | ≥40 - <50 min | 0·96 [0·92-1·01] | 0·130 |
|  | ≥30 - <40 min | 0·88 [0·83-0·92] | <·001 |
|  | ≥20 - <30 min | 0·81 [0·78-0·85] | <·001 |
|  | ≥10 - <20 min | 0·76 [0·73-0·79] | <·001 |
|  | <10 min | 0·83 [0·80-0·87] | <·001 |
|  | **Intervention of multiple coronary arteries** | 1·63 [1·58-1·68] | <·001 |
| Transport to hospital + in-hospital time to angiography | **Age (years)** |  |  |
|  | ≤ 59 | 1 |  |
|  | 60–74 | 2·01 [1·95-2·08] | <·001 |
|  | ≥ 75 | 4·44 [4·30-4·58] | <·001 |
|  | **Sex** |  |  |
|  | female | 1 |  |
|  | male | 0·90 [0·88-0·92] | <·001 |
|  | **Charlson comorbidity index** |  |  |
|  | 0 | 1 |  |
|  | 1 | 1·01 [0·98-1·03] | 0·537 |
|  | 2 | 1·12 [1·08-1·16] | <·001 |
|  | 3 | 1·44 [1·36-1·52] | <·001 |
|  | **Region of patient residence** |  |  |
|  | City | 1 |  |
|  | Urban area | 0·94 [0·90-0·97] | 0·006 |
|  | Rural area | 0·93 [0·88-0·97] | 0·047 |
|  | **Volume tertile hospital** |  |  |
|  | Low volume tertile hospital | 1 |  |
|  | Medium volume tertile hospital | 1·04 [0·98-1·10] | 0·191 |
|  | High volume tertile hospital | 1·07 [1·00-1·15] | 0·047 |
|  | **Performer** | 1·08 [1·05-1·12] | <·001 |
|  | **Time of day of admission** |  |  |
|  | 0.00 am – 5.59 am (ref.) | 1 |  |
|  | 6.00 am – 4.59 pm | 0·94 [0·90-0·98] | 0·001 |
|  | 5 pm – 11.59 pm | 1·13 [1·09-1·18] | <·001 |
|  | **Weekend admission** | 1·07 [1·04-1·09] | <·001 |
|  | **Transport to hospital + in-hospital time to angiography** |  | <·001 |
|  | ≥120 min | 1 |  |
|  | ≥90 - <120 min | 0·99 [0·95-1·04] | 0·648 |
|  | ≥80 - <90 min | 1·01 [0·95-1·06] | 0·812 |
|  | ≥70 - <80 min | 0·94 [0·89-0·99] | 0·025 |
|  | ≥60 - <70 min | 0·95 [0·90-1·00] | 0·040 |
|  | ≥50 - <60 min | 0·87 [0·83-0·92] | <·001 |
|  | ≥40 - <50 min | 0·84 [0·80-0·89] | <·001 |
|  | ≥30 - <40 min | 0·81 [0·77-0·85] | <·001 |
|  | <30 min | 0·84 [0·80-0·88] | <·001 |
|  | **Intervention of multiple coronary arteries** | 1·63 [1·58-1·68] | <·001 |

**Supp. Table 8: Results from a multivariable logistic regression model: Risk of in-hospital mortality.** Multivariable results stem from a multivariable mixed effect logistic regression model (generalized linear model: binomial, link function: logit, random effect: constant hospital identifier). Area under the curve: 0·70, 95% Confidence Interval, CI, 0·70-0·70 (In-hospital time to angiography), 0·70, CI, 0·70-0·70 (Transport to hospital + in-hospital time to angiography). The Charlson comorbidity index was obtained using the Stata plugin “charlson icd_sidediagnoses, index(10)”. 0: 0 points. 1: 1-2 points. 2: 3-4 points. 3: >4 points. Calibration plots in Supp. Figure 6, Forest plot of AUC in Supp. Figure 7. For the IHTA model, the maximum variance inflation factor was found for performer status with a variance inflation factor of 1·17. For the IHTA+TTH model, the maximum variance inflation factor was found for age groups with a variance inflation factor of 1·16.


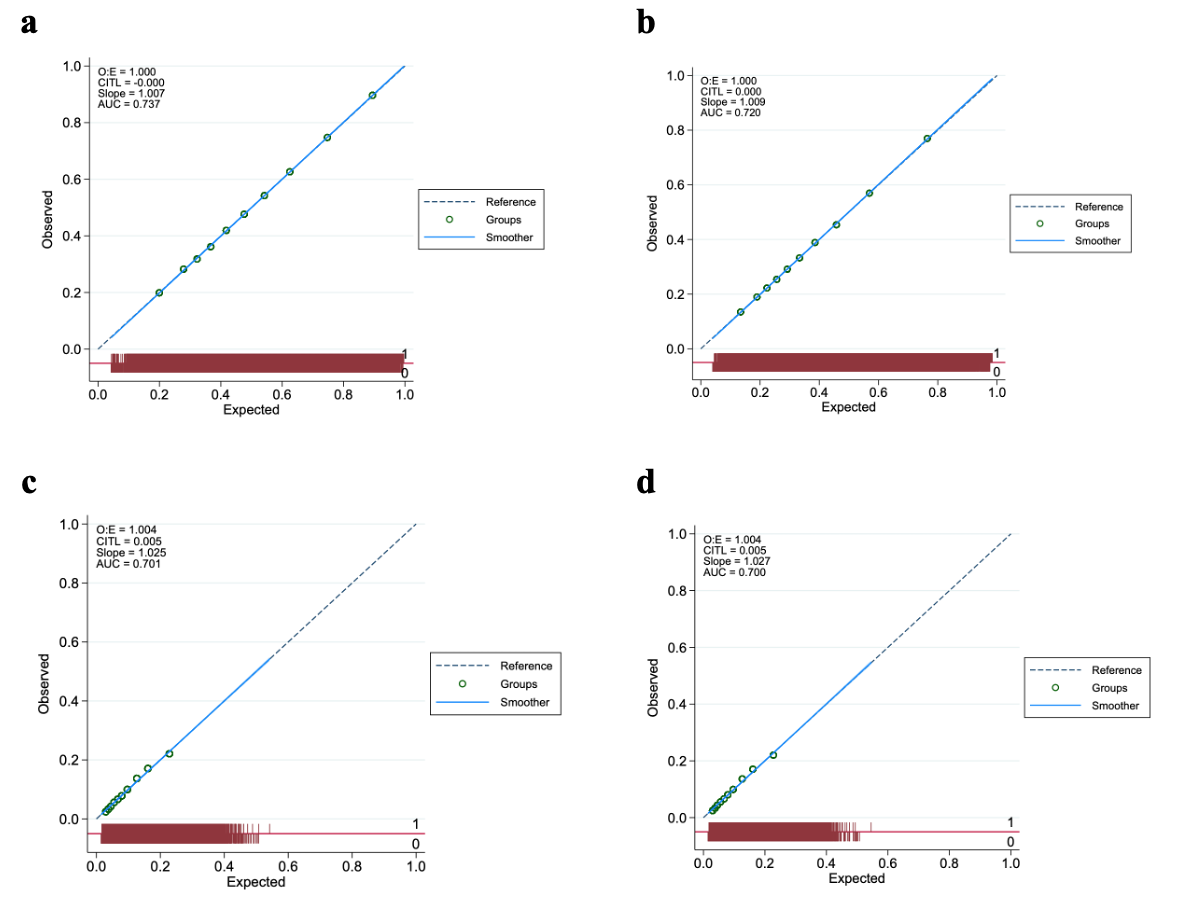


**Supp. Figure 6: Calibration plots. A** Logistic regression model with in-hospital time to angiography > 60 min as dependent variable as in Supp. Table 4. **B** Logistic regression model with transport to hospital + in-hospital time to angiography > 120 min as dependent variable as in Supp. Table 4. **C** Logistic regression model with in-hospital mortality as dependent variable, one independent variable being in-hospital time to angiography, as in Supp. Table 8. **D** Logistic regression model with in-hospital mortality as dependent variable, one independent variable being transport to hospital + in-hospital time to angiography, as in Supp. Table 8. Plots were produced using the Stata plugin “pmcalplot” for observed vs expected probabilities of the respective dependent variable of logistic regression models.

**Supp. Figure 7: Forest Plot: Area under the curve, AUC, for the logistic regression models.** Model 1 corresponds to **A** from Supp. Figure 6, i.e. logistic regression model with in-hospital time to angiography > 60 min as dependent variable as in Supp. Table 4. Model 2 corresponds to **B**, i.e. logistic regression model with transport to hospital + in-hospital time to angiography > 120 min as dependent variable as in Supp. Table 4. Model 3 corresponds to **C**, i.e. logistic regression model with in-hospital mortality as dependent variable, one independent variable being in-hospital time to angiography, as in Supp. Table 8. Model 4 corresponds to **D**, i.e. logistic regression model with in-hospital mortality as dependent variable, one independent variable being transport to hospital + in-hospital time to angiography, as in Supp. Table 8.

**Supp. Figure 8: Results of a post-hoc analysis of a multivariable logistic regression model of in-hospital mortality: margins of the year of admission. For assessment of the impact of the year of admission on in-hospital mortality, the Stata plugin “margins” was used to determine estimates of in-hospital mortality.** The logistic regression model with in-hospital mortality as dependent variable, one independent variable being in-hospital time to angiography, as in Supp. Table 8, was used, and year of admission was added as independent variable. Model performance and covariate estimators of the remaining variables remained largely unchanged. Odd’s Ratio, OR, for in-hospital mortality by year of admission (95% Confidence Interval, CI), 2012 as reference: 2013: 0·99 (0·94-1·05); 2014: 0·97 (0·92-1·03); 2015: 0·96 (0·91-1·02); 2016: 0·97 (0·92-1·03); 2017: 1·03 (0·97-1·09); 2018: 1·03 (0·97-1·09); 2019: 1·06 (1·01-1·12); 2020: 1·07 (1·02-1·13); 2021: 1·16 (1·10-1·22); 2022: 1·13 (1·07-1·19); 2023: 1·12 (1·06-1·18).
