# Supplementary material for: Time to coronary angiography and revascularization in 575,247 patients with STEMI from 2012 to 2023: a retrospective population-based cohort study
Source: Lancet Reg Health Eur. 2025 Dec 29;62:101576. doi: 10.1016/j.lanepe.2025.101576 (PMC12803846; doi:10.1016/j.lanepe.2025.101576)
Supplement: Translated Abstract [file mmc2.docx]

# Time to coronary angiography and revascularization in 575,247 patients with STEMI from 2012 to 2023 – A retrospective population-based cohort study

Short Title: Time to PPCI in STEMI 2012-2023

**Paulina E. Stürzebecher M.D. ^1 *^, Ulrich Laufs M.D. ^1 *^, Philip Baum M.D. ^2^, Johannes Diers M.D. ^3^, Armin Wiegering M.D. ^4,5,6^, and Konstantin Uttinger M.D. M.Sc. ^4,5,6^**

^1^ Klinik und Poliklinik für Kardiologie, Universitätsklinikum Leipzig, Leipzig, 04103, Germany

^2^ Department of Thoracic Surgery, Thoraxklinik at Heidelberg University Hospital, Röntgenstraße 1, 69126 Heidelberg, Germany

^3^ Marienkrankenhaus Hamburg, Alfredstraße 9, 22087 Hamburg, Germany

^4^ Department of General, Visceral, Transplant and Thoracic Surgery at Frankfurt University Hospital, Goethe University, Frankfurt am Main, Germany

^5^ Frankfurt Cancer Institute, Georg-Speyer-Haus, Paul-Ehrlich-Str. 42-44, 60596 Frankfurt am Main, Germany

^6^ University Cancer Center Frankfurt (UCT), Frankfurt University Hospital, Goethe University, Frankfurt am Main, Germany

Correspondence to: Dr. Konstantin Uttinger, Department of General, Visceral, Transplant and Thoracic Surgery, Frankfurt University Hospital, Goethe University Frankfurt/Main, Theodor-Stern-Kai 7, 60590 Frankfurt/Main, Germany, e-mail: [konstantin@uttinger.com](mailto:konstantin@uttinger.com)

**Zusammenfassung**

**Hintergrund:** Eine rasche primäre perkutane Koronarintervention (PPCI) bei Patienten mit ST-Hebungsinfarkt (STEMI) reduziert die Krankenhaus- und Langzeitmortalität. Diese Studie analysiert die Zeitintervalle bis zur PPCI bei STEMI, Risikofaktoren für Verzögerungen der PPCI sowie die Krankenhausmortalität im Zeitraum von 2012 bis 2023.

**Methoden:** Es handelt sich um eine retrospektive, bevölkerungsbasierte Analyse von Krankenhausabrechnungsdaten erwachsener STEMI-Patienten, die in Deutschland eine PPCI erhielten. Die Transportzeit zum Krankenhaus (TTH) wurde mithilfe geografischer Fahrzeitschätzungen bestimmt. Die innerklinische Zeit bis zur Angiographie (IHTA) wurde anhand der Zeitkodierung der PPCI in den Patientendaten berechnet.

**Ergebnisse:** Insgesamt wurden 575.247 Patienten analysiert. Das mediane Alter betrug 64 Jahre, 28,5% (164.016) waren weiblich. Der Anteil der Patienten mit einer IHTA ≤ 60 Minuten stieg von 44,5% (22.240/49.965) im Jahr 2012 auf 57,7% (24.434/42.356) im Jahr 2023, mit einer Verbesserung der kombinierten TTH+IHTA ≤ 120 Minuten (von 56,6%, 28.280/49.965, im Jahr 2012 auf 70,2%, 29.734/42.356, im Jahr 2023). Die mediane IHTA verbesserte sich von 73,1 Minuten (IQR 25,2–186,6) im Jahr 2012 auf 46,4 Minuten (IQR 17,5–111,6) im Jahr 2023, während die TTH stabil blieb (11,4–11,9 Minuten).
Risikofaktoren für eine IHTA > 60 Minuten waren höheres Alter, weibliches Geschlecht, Komorbiditäten, Aufnahme außerhalb der Regelarbeitszeiten sowie Behandlung in Krankenhäusern mit geringem Behandlungsvolumen. Die Krankenhausmortalität stieg über den Beobachtungszeitraum (8,8%, 4.406/49.965, in 2012, 10,4%, 4.822/46.203, in 2021, 10,1%, 4.272/42.356, in 2023) parallel zu einem Anstieg des Patientenalters und der Komorbiditäten. Risikofaktoren für die Krankenhausmortalität umfassten weibliches Geschlecht, höheres Alter, Komorbiditäten, Behandlung in Hochvolumenkliniken, Interventionen an mehreren Koronararterien, Wochenendaufnahme sowie Vorstellung außerhalb der Regelarbeitszeiten. Eine IHTA < 40 Minuten (Referenz: 90–120 Minuten) und eine TTH+IHTA < 80 Minuten (Referenz: ≥ 120 Minuten) reduzierten das Sterberisiko.

**Interpretation:** Die Kombination von Krankenhausabrechnungsdaten mit geografischer Fahrzeitschätzung ermöglicht ein Benchmarking sowohl präklinischer als auch innerklinischer Verzögerungen in der STEMI-Versorgung. Die innerklinischen Verzögerungen nahmen zwischen 2012 und 2023 ab. Wichtige Ansatzpunkte zur weiteren Reduktion von Zeitverzögerungen und STEMI-bedingter Mortalität liegen in der Verbesserung der Behandlungszeiten außerhalb der Regelarbeitszeiten sowie in einem besonderen Fokus auf Frauen, ältere Patienten und Personen mit Begleiterkrankungen.
